# Prevalence and characterization of breakthrough pain in patients with cancer in Spain: the CARPE-DIO study

**DOI:** 10.1038/s41598-019-54195-x

**Published:** 2019-11-27

**Authors:** Concepción Pérez-Hernández, Ana Blasco, Álvaro Gándara, Ana Mañas, Manuel Jesús Rodríguez-López, Vicente Martínez, Alonso Fernandez-Nistal, Carmen Montoto

**Affiliations:** 1 Pain Unit, Hospital Universitario La Princesa, Madrid, Spain; 2 Medical Oncology Department, Hospital General Universitario de Valencia, CIBERONC, Valencia, Spain; 3 Palliative Care Unit, Hospital Universitario Fundación Jiménez Díaz, Madrid, Spain; 4 Radiation Oncology Department, Hospital Universitario La Paz, Madrid, Spain; 5 Pain Unit, Hospital Regional Universitario Carlos Haya, Málaga, Spain; 6 Medical Department, Takeda Farmacéutica España, Madrid, Spain

**Keywords:** Oncology, Pain

## Abstract

We aimed to evaluate the prevalence, characteristics and impact of breakthrough pain (BTP) in patients with cancer attending the main specialties involved in the diagnosis and management of BTP in Spain using a multicenter, observational, cross-sectional, multidisciplinary study. Investigators had to record all patients seen at the clinic during 1 month, determine whether the patients had cancer pain, and apply the Davies algorithm to ascertain whether the patients were suffering from BTP. Of the 3,765 patients with cancer, 1,117 (30%) had cancer-related pain, and of these patients, 539 had BTP (48%, 95%CI:45–51). The highest prevalence was found in patients from palliative care (61%, 95%CI:54–68), and the lowest was found in those from hematology (25%, 95%CI:20–31). Prevalence varied also according to sex and type of tumor. According to the Alberta Breakthrough Pain Assessment Tool duration, timing, frequency, location, severity, quality, causes, and predictability of the BTP varied greatly among these patients. BTP was moderate (Brief Pain Inventory [BPI]-severity median score = 5.3), and pain interference was moderate (BPI-interference median score = 6.1) with a greater interference with normal work, general activity, and enjoyment of life. Patients with BTP showed a mean ± standard deviation score of 28.5 ± 8.0 and 36.9 ± 9.5 in the physical and mental component, respectively, of the SF-12 questionnaire. In conclusion, prevalence of BTP among patients exhibiting cancer-related pain is high. Clinical presentation is heterogeneous, and therefore, BTP cannot be considered as a single entity. However, uniformly BTP has an important impact on a patient’s functionality, which supports the need for early detection and treatment.

## Introduction

Breakthrough pain (BTP) has been defined as a “transient exacerbation of pain that occurs either spontaneously, or in relation to a specific predictable or unpredictable trigger despite relatively stable and adequately controlled background pain”^1^. A Spanish consensus on BTP in patients with cancer considers that treatment with opioids is a requirement for its diagnosis^2^. However, this condition is far from a uniform clinical entity, and even experts in this area consider that transient exacerbation of pain can occur without background pain or when background pain is uncontrolled or regardless of opioid treatment^3,4^.

Breakthrough pain (BTP) is a common problem encountered in clinical practice in patients with cancer, has a heterogeneous clinical presentation, and is associated with an important impact on the patient, family, caregivers and society. The prevalence of BTP varies greatly depending on the study, especially on the definition and the setting^5^. It should be noted that there are several definitions and methods of classifying BTP as well as several tools for screening and/or diagnosing this condition^6^. Deandrea *et al*.^7^ in a systematic review of 19 studies, found the lowest prevalence (40%) of BTP in studies conducted in outpatients and the highest prevalence in those conducted in a hospice setting (≈80%); they estimated an overall pooled prevalence of 59%.

The clinical picture of BTP varies greatly from patient to patient. In a European study conducted in 1,000 patients with cancer, 44% had incident pain, 41.5% had spontaneous pain and 14.5% had a combination of both forms of presentation^8^. In that study, the median number of episodes of BTP ranged from 1/month to 24/day, the median time to peak intensity ranged from less than 1 minute to 240 minutes, and the pain intensity was moderate in 34% of patients and severe in 62% of patients^8^. Similar heterogeneous presentation of BTP has been reported in surveys conducted in Canada^9^ and Norway^10^, and the clinical picture appears very similar on both sides of the Atlantic^11^. Age, type of cancer, performance status, background pain intensity, and mechanism are among the factors affecting the clinical presentation of BTP^12^. Breakthrough cancer pain has an important impact on the individual in terms of the impairment of daily life activities and quality of life. Most patients with BTP report that pain stops them from doing something^8^. In patients with advanced cancer, those exhibiting BTP had greater impairment in most dimensions of quality of life than those without BTP^13^. BTP not only interferes with general activities but also has a negative impact on pain management; in a study conducted on 258 patients who had received opioids, the presence of BTP was associated with a lower likelihood of achieving personalized pain goals^14^. Finally, BTP is responsible for a substantial economic burden due to the increased utilization of healthcare services, including hospitalization^15^.

Information on the prevalence and clinical characteristics of BTP in Spain is very limited. In a study conducted on a sample of patients with advanced cancer attending the palliative care outpatient clinic of a university hospital in Lleida, Canal-Sotelo *et al*. recently reported a prevalence of BTP of 39% and great variability in the type of presentation using a retrospective evaluation with the Davies algorithm^16^. Very recently, Camps *et al*. reported the results of a survey conducted by 108 medical oncologists throughout Spain and found that 493 (91%) of the 540 patients evaluated exhibited BTP also using the Davies algorithm; more importantly, over 40% of the cases of BTP had not been previously detected by the medical oncologist^17^.

The primary objective of this study was to evaluate the prevalence of BTP in patients with cancer attending the main specialties involved in the diagnosis and management of BTP in Spain (namely, medical oncology, radiation oncology, hematology, pain units, and palliative care units). Secondary objectives included evaluating the 1-month cumulative incidence of BTP, describing the clinical characteristics of patients with undiagnosed BTP, and evaluating the impacts on daily activities and quality of life.

## Patients and Methods

This was a multicenter, observational, cross-sectional, multidisciplinary study conducted in outpatients of the medical oncology, radiation oncology and hematology departments as well as in those attending pain and palliative care units from November 2016 to April 2018. The study was approved by the Ethics Committee of the Hospital La Princesa (Madrid, Spain) and conducted following the principles of the Helsinki Declaration.

The study was undertaken in two steps. In the first step, to evaluate the prevalence of BTP, participant investigators had to record all patients seen at the clinic during 1 month, determine whether the patients had cancer pain, and apply the Davies algorithm to ascertain whether the patients were suffering from BTP. In addition, investigators recorded information on age, sex, Karnofsky performance status, type of tumor and stage. In the second step, to analyze the characteristics of patients with cancer and BTP, participant investigators had to recruit the first two patients of each day who met the selection criteria up to a maximum of 10 patients. To be included in this second step, patients had to be 18 years or older, present oncologic pain adequately controlled with opioids, meet the criteria of BTP according to the Davies algorithm, not be under treatment for BTP, and give written informed consent. To meet Davies’ criteria^1^, the patient had to exhibit pain for ≥12 hours/day during the previous week, or pain had to be present if not taking analgesia; the background pain had to be rated as none or mild but not moderate or severe for ≥12 hours/day during the previous week; and the patient had to present transient exacerbations of pain. Patients were excluded if they had a severe mental disorder or had a medical condition or other situation that in the investigator’s judgment greatly interfered with data collection.

In patients who met the selection criteria, we recorded information on age, sex, type of cancer and tumor stage at the time of diagnosis, previous treatment with surgery, chemotherapy and/or radiotherapy, current tumor stage and treatment, and the presence of the following signs or symptoms: mucositis, nausea/vomiting, constipation, bone fractures, xerostomia, peripheral neuropathy or other. The presence of comorbidities was evaluated using a checklist including the following entities: ischemic heart disease, heart failure, peripheral arterial disease, cerebrovascular disease, dementia, Parkinson’s disease, hemiplegia, chronic respiratory disease, connective tissue disease, gastroduodenal ulcers, mild chronic hepatic disease, moderate/severe chronic hepatic disease, diabetes, diabetes with target organ damage, moderate/severe chronic renal disease, AIDS, and second neoplasia. The following characteristics of the BTP were evaluated: iatrogenic origin, the mechanism (nociceptive, neuropathic or both), triggers (incidental or spontaneous), location and type. According to the intensity and duration and based on the anamnesis, pain had to be classified as type I (pain with a short duration and high intensity that begins and ends suddenly), type II (pain with a long duration and moderate-high intensity that begins and ends gradually), type III (successive pain peaks of gradually decreasing intensity) or type IV (pain that starts gradually, reaches a maximum peak and is maintained for a period of time, gradually decreasing thereafter). Finally, the following evaluation tools were applied: the Brief Pain Inventory (BPI)^18^, the Alberta Breakthrough Pain Assessment Tool^19,20^, the Karnofsky scale^21^, and the SF-12 questionnaire^22,23^. The participant investigators did not received any training on the hetero-administered evaluation tools.

The Alberta Breakthrough Pain Assessment Tool (ABPAT) consists of a patient self-reporting section (15 questions) that assesses the relationship of pain flares to background pain, further probes for details about sources of relief, and enquires about the timing, frequency, location, severity, quality, causes, and predictability of the BTP; the last four items of the tool were not included since they are related to the treatment of BTP. The ABPAT also has two questions to be answered by the physician or the nurse concerning the etiology and pathophysiology of pain. The ABPAT has been validated in patients with cancer pain who present with BTP^19,20^.

### Statistical analysis

With a precision of ±1%, a confidence level of 95%, and an expected frequency of 50% of patients presenting with BTP (a conservative estimate for a common but unknown prevalence), a minimum sample of 13,200 patients seen at the investigators’ clinics was needed.

Quantitative variables are described with the mean and the standard deviation (SD) or, if needed, with the median and the interquartile range (IQR). Qualitative variables are described with absolute and relative frequencies.

To calculate the prevalence of BTP, we included the number of patients with BTP as defined with Davies’ criteria in the numerator and the number of patients with oncologic pain adequately controlled with opioids in the denominator; the corresponding 95% confidence interval was also calculated. The prevalence of BTP is also presented by age, sex, specialty, type of tumor, tumor stage, and performance status. The cumulative 1-month incidence of undiagnosed BTP was calculated in the same way but using new cases of BTP (i.e., those not previously diagnosed) in the numerator. A subgroup analysis according to age (<70 and ≥70 years) was also performed.

All analyses were performed using IBM SPSS Statistics version 22.

### Ethical approval

The study was approved by the Ethics Committee of the Hospital La Princesa (Madrid, Spain).

### Informed consent

Written informed consent was obtained from every subject.

## Results

### Patient disposition and characteristics

A total of 3,765 patients were seen at 32 sites by 43 specialists from pain units (n = 15), radiotherapeutic oncology (n = 9), medical oncology (n = 7), hematology (n = 6) and palliative care (n = 6). Patients were predominantly male (n = 1947, 52%), aged below 70 years (n = 2,249, 60%) and with a performance status of 80 or below in 1,841 (49%) of the 3,751 patients with available information. The most frequent types of tumors were multiple myeloma (n = 740, 20%) and breast cancer (n = 618, 16%), and most patients had stage III/IV tumors (n = 2,041, 65%).

### Prevalence and cumulative incidence of breakthrough pain

**Figure 1:**
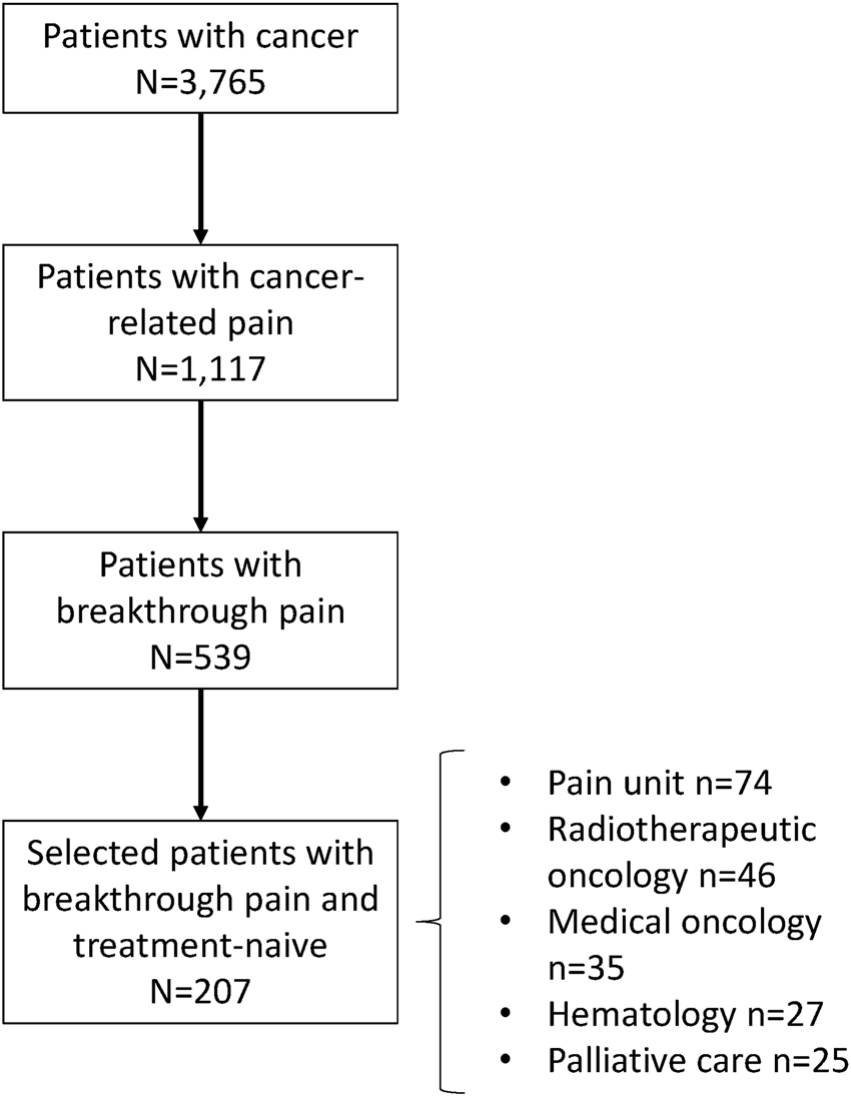
Patient disposition.

**Figure 2:**
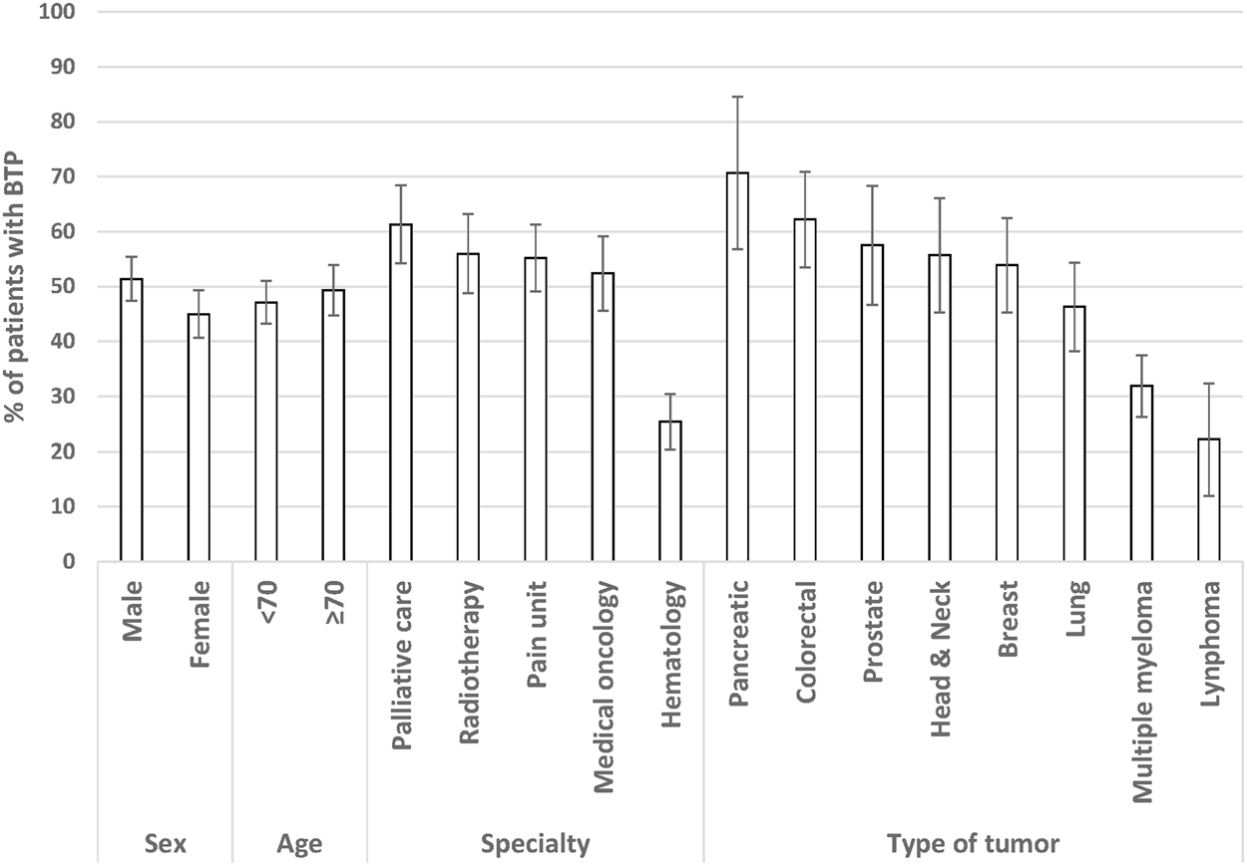
Prevalence of breakthrough pain by age, sex, specialty and type of tumor. Vertical bars correspond to 95% confidence intervals. BTP, breakthrough pain.

Of the 3,765 patients with cancer, 1,117 (30%) had cancer-related pain, and of these patients, 539 had BTP (48%, 95% CI: 45 to 51) (Fig. 1). The prevalence of BTP was higher in males than in females (51% vs. 45%) (Fig. 2). The highest prevalence was found in patients from palliative care (61%, 95% CI: 54 to 68), and the lowest was found in those from hematology (25%, 95% CI: 20 to 31); by the type of tumor, the highest prevalence was found in patients with pancreatic cancer (71%, 95% CI: 57 to 85) and colorectal cancer (62%, 95% CI: 54 to 71), and the lowest prevalence was found among patients with multiple myeloma (32%, 95% CI: 26 to 38) and lymphoma (22%, 95% CI: 12 to 33) (Fig. 2). The prevalence of BTP increased as the Karnofsky performance status decreased, with the lowest prevalence in those with a performance status of 90 (38%) and the highest in those with a performance status of 30 (80%). The prevalence also increased as the tumor stage was more advanced and ranged from 32% in patients with stage I to 57% in those with stage IV disease. The cumulative 1-month incidence of undiagnosed BTP was 19% (95% CI: 16 to 23).

### Characteristics of patients with breakthrough pain who were untreated

Of the 539 patients with BTP, we recruited 207 consecutive patients who were not receiving treatment for BTP, mostly from pain units (36%), radiotherapeutic oncology (22%) and medical oncology (17%) (Fig. 1). Patients were predominantly male (56%), aged below 70 years (62%) and with a performance status of 80 or below (88%). The most frequent types of tumors were breast (18%) and lung cancer (15%), and most patients had stage IV disease (65%) (Table 1).

**Table 1:** Characteristics of patients with breakthrough pain who were treatment-naïve*.

Characteristic	N	
Sex (female), n (%)	207	90 (43.5)
Age	206	
Mean (SD)		65 (12)
≥70 years, n (%)		78 (37.9)
Race (Caucasian), n (%)	207	199 (96.1)
Karnofsky (≤80), n (%)	202	178 (88.1)
Most frequent (≥5%) comorbidities, n (%)	207	
Diabetes		41 (19.8)
Chronic pulmonary disease		30 (14.5)
Ischemic heart disease		18 (8.7)
Peripheral arterial disease		17 (8.2)
Secondary neoplasm/other tumor		14 (6.8)
Chronic kidney disease		11 (5.3)
Most frequent (≥5%) primary cancer diagnosis, n (%)	207	
Breast		37 (17.9)
Lung		31 (15.0)
Multiple myeloma		27 (13.0)
Head & Neck		22 (10.6)
Colorectal		20 (9.7)
Prostate		18 (8.7)
Stage at the study entry, n (%)	184	
I		10 (5.4)
II		25 (13.6)
III		30 (16.3)
IV		119 (64.7)
Current treatment, n (%)	207	
Surgery		18 (8.7)
Chemotherapy		107 (51.7)
Radiotherapy		74 (35.7)
Most frequent (10%) signs and symptoms, n (%)		
Constipation	207	50 (24.2)
Xerostomia		38 (18.4)
Peripheral neuropathy		29 (14.0)
Nausea/vomiting		28 (13.5)
Mucositis		24 (11.6)
Fracture		13 (6.3)
Other		32 (15.5)

^*^For breakthrough pain.

SD, standard deviation.

**Figure 3:**
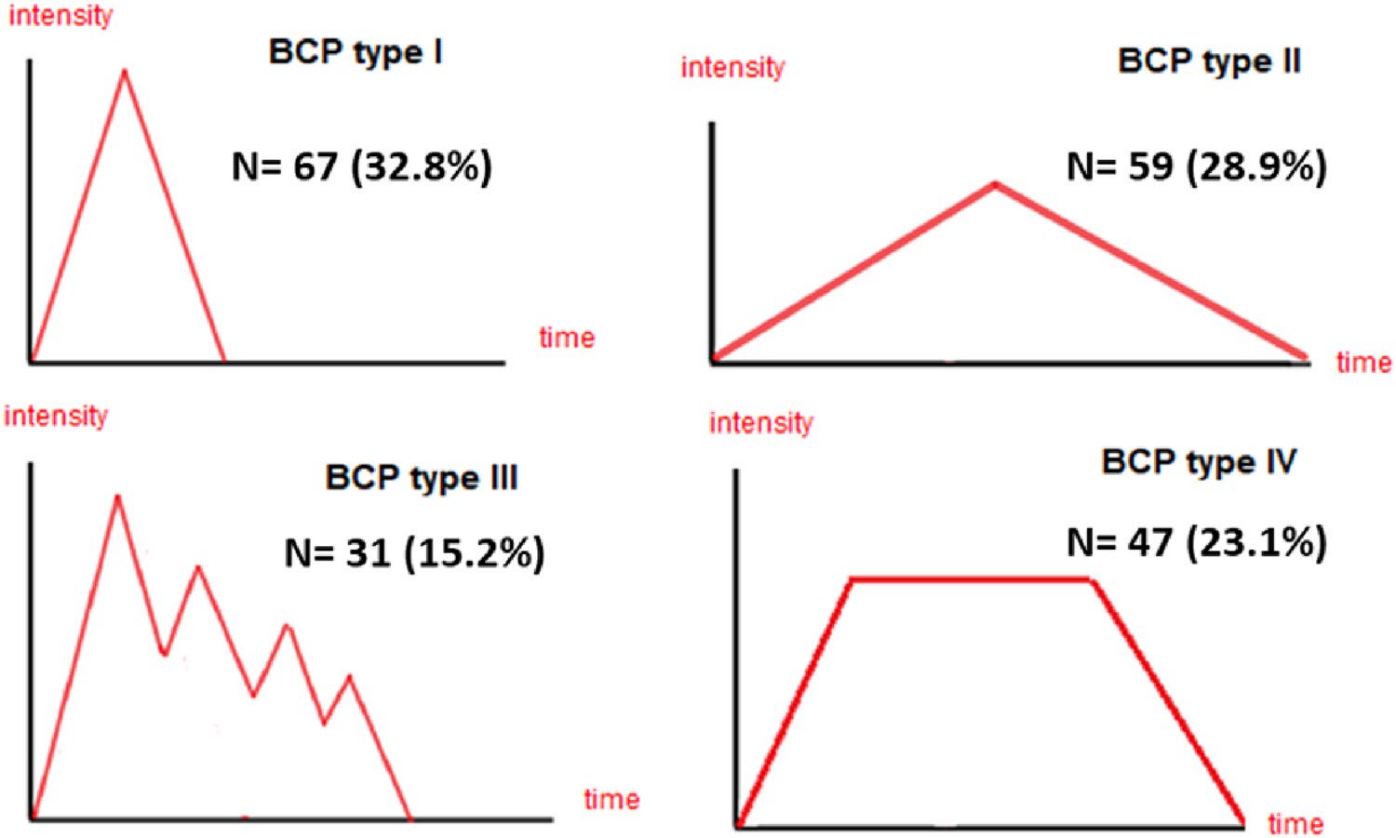
Types of breakthrough pain according to intensity and duration.

Breakthrough pain was spontaneous in 88/206 (43%) patients and incidental in 118/206 (57%). Among patients with incidental pain who were further categorized depending on the trigger (n = 117), BTP was volitional in 78 (67%), nonvolitional in 29 (25%) and procedural in 10 (9%). Depending on the intensity and duration (Fig. 3), the most frequent types of BTP were type I (33%) and type II (29%). Patients ≥70 years showed overlapping results (data not shown).

**Figure 4:**
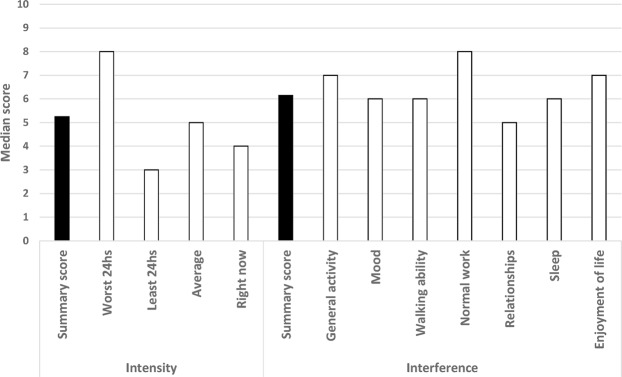
Pain intensity and interference in patients with breakthrough pain as evaluated with the Brief Pain Inventory.

According to the ABPAT (Table 2), the pain was most frequently described as a brief flare-up to baseline pain (53%), of severe intensity (83%), located in a wide array of locations (most frequently lumbar [20%]) and qualitatively described as stabbing (58%). The latency to peak intensity was usually 10 minutes or less (57%), lasting 30 minutes or less (56%), and was somewhat unpredictable in 37% of the patients. The pain was considered to be related to the site of active cancer in 78% of the patients and was most frequently categorized as nociceptive (somatic in 56% of the patients and visceral in 16% of the patients). When evaluated with the BPI, although the overall intensity of BTP was moderate (a median score of 5.3 in the pain severity score), at worst, BTP was severe with a median score of 8 (Fig. 4). Similarly, pain interference was moderate (a median score of 6.1 in the summary score of the BPI) with a greater interference with normal work, general activity, and enjoyment of life (Fig. 4). Patients with BTP showed a mean (SD) score of 28.5 (8.0) in the physical component and 36.9 (9.5) in the mental component of the quality of life as measured with the SF-12 scale. The results of the subgroup of patients ≥70 years regarding the ABPAT, BPI and SF-12 scales did not differ to a relevant extent from those of the total sample (data not shown).

**Table 2:** Pain characterization with the Alberta Breakthrough Pain Assessment.

Question	N	
**Completed by the patients**		
*Q1. Relationship to baseline pain*	190	
Brief flare-up of baseline pain		101 (53.2)
Different from baseline pain		69 (36.3)
Not sure		20 (10.5)
*Q2a. Last time experienced*	194	
Today		112 (57.7)
Yesterday		64 (33.0)
Before then		18 (9.3)
*Q3a. Frequency*	182	
Mean (SD)		2.9 (2.1)
*Q3b. Frequency*	182	
Usual		112 (62.6)
Better		15 (8.4)
Worse		52 (29.1)
*Q4a. Intensity of pain at peak*	195	
Mean (SD)		8.5 (1.5)
*Q4b. Intensity of pain at peak*	194	
Mild		3 (1.5)
Moderate		31 (16.0)
Severe		160 (82.5)
*Q5. Location (most frequent -* ≥ *5%)*	195	
Lumbar		39 (20.0)
Hips		13 (6.7)
Abdominal		13 (6.7)
Back		12 (6.2)
Thoracolumbar		10 (5.1)
Thoracic		10 (5.1)
*Q6. Quality (Those present in* ≥ * 20%)*	195	
Stabbing		113 (57.9)
Splitting		58 (29.7)
Sharp		57 (29.2)
Punishing-Cruel		53 (27.2)
Heavy		52 (26.7)
Hot-Burning		50 (25.6)
Fearful		42 (21.5)
*Q7. Time from onset to peak intensity*	195	
More than 0 and up to 10 minutes		112 (57.4)
More than 10 and up to 30 minutes		47 (24.1)
More than 30 minutes		16 (8,2)
It is hard to say exactly when it started		20 (10.3)
*Q8. Time from onset [take medication] to end of episode*	192	
More than 0 and up to 10 minutes		36 (18.8)
More than 10 and up to 30 minutes		72 (37.5)
More than 30 minutes		64 (33.3)
I am not on any breakthrough pain medication		20 (10.4)
*Q9. Cause(s) (triggers) (Those present in* ≥ * 20%)*	195	
Movement in bed		65 (33.3)
Walking		62 (31.8)
Standing		58 (29.7)
Sitting		38 (19.5)
Coughing		38 (19.5)
*Q10. Predictability*	195	
I can never predict when it will occur		38 (19.5)
I can rarely predict when it will occur		34 (17.4)
I can sometimes predict when it will occur		40 (20.5)
I can often predict when it will occur		59 (30.3)
I can always predict when it will occur		24 (12.3)
*Q11. General relief (those present in ≈20% or more patients)*	195	
Lying		73 (37.4)
Use of scheduled pain medication		38 (19.5)
Unsure		38 (19.5)
**Completed by nurses/physicians**		
*Q1. Etiology of breakthrough pain*	186	
Pain related to the site of active cancer		145 (78.0)
Pain related to the whole body or cancer’s systemic effects		11 (5.9)
Pain related to anticancer treatment		18 (9.7)
Pain caused by a concurrent disorder		9 (4.8)
Unknown or uncertain at this time		3 (1.6)
*Q2. Inferred pathophysiology of breakthrough pain*	186	
Somatic nociceptive		104 (55.9)
Visceral nociceptive		30 (16.1)
Neuropathic		37 (19.9)
Unknown or uncertain at this time		15 (8.1)

All figures are number of patients and percentage except otherwise indicated.

N, number of evaluable patients; SD, standard deviation.

In a *post hoc* analysis, type III BTP showed greater intensity (median score of 6.6 in the BPI) and interference (median score of 7.9) as well as the worst quality of life (mean [SD] scores of 25.9 [4.5] in the physical component and 31.0 [8.7] in the mental component of the SF-12); the mean Karnofsky score was significantly lower in patients with type III BTP (58 in type III BTP vs. 65–69 in the remaining types of BTP).

## Discussion

This study across several specialties dealing with cancer shows that breakthrough pain is present in almost half of the patients with cancer-related pain and is more common in patients with nonhematologic tumors, especially those with pancreatic or colorectal cancer. The characteristics of treatment-naïve breakthrough pain are heterogeneous, although in a majority of patients it is nociceptive, severe and associated with a relevant impairment of daily life.

We found that 48% of patients with cancer-related pain had BTP. This figure is consistent with the results of a retrospective study recently conducted in a single center in Spain, which, using the Davies algorithm in outpatients from the palliative care department, found a prevalence of BTP of 39%^16^. However, other studies conducted in Spain have found higher prevalence rates. Thus, in a recent study conducted by 108 medical oncologists, Camps *et al*. reported a prevalence of 91% of BTP using the Davies algorithm^17^. We think that the selection of patients in that study could explain, at least in part, this high prevalence of BTP; these authors selected patients who, before the start of the study, had been treated by the participant oncologists as patients suffering some kind of cancer-related pain^17^. In contrast, we based our selection on all patients with cancer seen at the participant sites, except for patients seen at pain units, regardless of whether they had cancer-related pain. The authors of the validation of BPI in cancer patients in Spain also found a higher prevalence of BTP (70%)^18^. However, they also selected patients who already had cancer-related pain and diagnosis of BTP was not based on the Davies algorithm but on the physician evaluation^18^. Overall, we think that, bearing in mind the patient selection process, the figures reported by Canal-Sotelo *et al*.^16^ in their unicenter study and our figures probably better reflect the quantitative dimension of the problem in Spain. Our results are also consistent with those reported by Greco *et al*.^24^ in Italy, who found a prevalence of BTP of 52% using the Edmonton Staging System in patients with cancer and pain. They are also consistent with those of a European survey that, similar to us, was conducted by several specialists dealing with cancer-related pain (namely, general oncology, medical, palliative care and hospice units) and found that 289 (42%) out of the 682 patients with cancer pain had BTP using the definition of BTP included in the ABPAT^13^. Finally, our prevalence figures are within the range reported by Andrea *et al*. in their systematic review of the literature^7^.

According to our results, the prevalence of BTP varies depending on the type of tumor, with the highest prevalence being in patients with pancreatic pain and colorectal cancer and the lowest in patients with hematological malignancies. The type of tumor may influence some clinical characteristics of BTP^12^, but we are not aware of similar studies reporting the prevalence of BTP according to the type of tumor, and therefore, our results could not be put into perspective in this regard. Consistent with the lowest prevalence among patients with hematologic malignancies, the prevalence of BTP was much lower in hematology (25%) than in other specialties involved in our study, especially among patients treated in the palliative care department (61%). Greco *et al*.^24^ in a study conducted in Italy also found a higher prevalence in patients from palliative care, pain units and hospices compared to those treated in oncology departments/centers.

In a systematic review of the literature, Deandrea *et al*.^7^ also found that the prevalence of BTP is higher in patients from palliative care and those managed in a hospice. This latter study also identified several other factors affecting the prevalence of BTP; thus, prevalence was higher in older patients, males, and those with advanced disease^7^. Our results are somewhat consistent with this pattern, although there were only slight differences in prevalence rates between males and females and between patients aged ≥70 years and those <70 years. The prevalence of BTP also varies with performance status and tumor stage, but regardless of these characteristics, the prevalence is high in every subgroup. All these data support that, as recommended by the experts^4^, all patients with cancer-related pain, regardless of tumor stage or functional status, should be screened for the presence of BTP.

The type of pain according to its relation to trigger events (spontaneous vs. incidental) was evenly distributed among patients with BTP. Almost overlapping results were found in the study conducted by Davies *et al*. in 13 European countries^8^, and the results from Europe do not seem to greatly differ from those from Canada^11^. Consistent with previous studies^8,11,12,25,26^, we found an important variability regarding time to peak intensity and pain duration. More uniform are the results regarding the underlying mechanism of BTP and the peak intensity; our results and the literature consistently show that most patients exhibit nociceptive pain with severe intensity and that BTP was triggered by some kind of movement^8,20,25,26^. According to our results and those from other authors^12,20,25,26^, in a substantial proportion of patients −37% in our study and over 50% in other studies- BTP is fairly unpredictable. When categorized by its intensity and duration (types I to IV), we found a relevant proportion of patients in each category ranging from 15% of patients exhibiting type III BTP to 33% of those exhibiting type I BTP. Regardless of the characteristics of BTP, it has an important impact on the individual in terms of interferences with work and daily activities; our results using the BPI overlap those of Davies’ in the European survey^8^. We also found an important impairment of the quality of life as evaluated with the SF-12 questionnaire; however, although we know that BTP contributes to the deterioration of the quality of life in patients with cancer^13^, unfortunately, we are not able to evaluate the contribution of BTP to this impairment. Interestingly, the characteristics of BTP seem to be associated with a differential impact on quality of life; thus, we found that type III BTP (i.e., that characterized by successive pain peaks of gradually decreasing intensity) was associated with greater intensity and interference with daily activities and worst quality of life. These results suggest that the categorization of pain according to intensity and duration may be useful for detecting patients suffering from a greater impact of pain and could provide some orientation for pain management. However, our cross-sectional design does not allow us to establish a causal relationship for this association, and further research is needed in this regard.

Study limitations include the already mentioned cross-sectional design for some evaluations and the sample size, which was far below the one estimated in our sample size calculation. This latter issue should be considered in the interpretation of the prevalence results of the several subgroups analyzed due to the lack of precision of the estimates. Overall, the prevalence of cancer-related pain in our study (i.e. 33%) could be considered low. A systematic review of 122 studies found that the prevalence of pain in patients with cancer ranges from 39% to 66% depending on the cancer treatment status^27^. We think that the apparently low prevalence of cancer pain in our study could be explained in part because in our study patients were recruited from several sources, some of them not commonly included in previous prevalence studies (e.g. pain units, radiation oncology, hematology); other potential explanations are that we excluded patients with pain not secondary to the disease, and that a substantial number of recruited patients had hematological malignancies. Another limitation is that although we intend to include patients with disease-related pain, it is possible that participant investigators have included some patients with iatrogenic pain (e.g. chemotherapy- or radiotherapy-induced neuropathic pain). We think that the need of being stabilized under treatment with opiods –a treatment uncommonly used for these conditions- limits the possibility of these erroneous inclusions. Our categorization of pain according to pain duration and severity was based on ad-hoc evaluation tool and thus requires further replication and validation. Finally, this study was sponsored by a pharmaceutical company and this could have introduced a bias.

In conclusion, our study confirms in the Spanish setting the high prevalence of breakthrough pain among patients exhibiting cancer-related pain. Although the prevalence of breakthrough pain varies according to several factors, including type of cancer, tumor stage and performance status, regardless of these characteristics, it is present in a substantial proportion of patients, indicating that this problem should be investigated in every patient with cancer pain. Clinical presentation is heterogeneous, and therefore, breakthrough pain cannot be considered as a single entity. However, uniform breakthrough pain has an important impact on a patient’s functionality, which supports the need for early detection and treatment, especially considering that some surveys indicate that breakthrough pain is frequently undetected^17^ and undertreated^24,28^, and our results indicate that over 40% of patients are able to foretell the occurrence of BTP episodes.

## Data Availability

The datasets used and/or analyzed during the current study are available from the corresponding author on reasonable request
